# Bioremediation of malachite green dye toxicity under optimized conditions by *Rhodotorula mucilaginosa* AUMC13567

**DOI:** 10.1186/s12896-025-00977-3

**Published:** 2025-05-17

**Authors:** Maysa M. Ali, Somaya Nassar, Nivien Allam Nafady, Eman Mostafa Mohamed

**Affiliations:** Faculty of Science, Botany and Microbiology Department, Assiut University, Assiut, Egypt

**Keywords:** Cytotoxicity, Decolorization, Malachite green, Wastewater, Yeast

## Abstract

The escalating impacts of human urbanization and rapid industrial development have resulted in a growing global demand for synthetic textile dyes. Malachite green (MG) dye is a hazardous chromatic compound predominantly utilized as a textile dye, exhibiting a strong affinity for the substrate to which it is applied. When diluted, it serves as antimicrobial agent for fungal and bacterial diseases in fish farms. However, the disposal of textile dyeing wastes pose significant environmental challenges, contaminating soil, surface water, and adversely affecting human health and aquatic life. *R. mucilaginosa* exhibits robust growth and resistance capabilities even under conditions of nutrient deficiency or external stress. This study focuses on optimizing the malachite green dye degradation ability of the yeast strain *Rhodotorula mucilaginosa* AUMC13567. The results reveal that the optimal conditions for maximum decolorization which was 100% at 50 mg/L dye concentration involve cultivating *R. mucilaginosa* on medium comprising distilled water with 3% glucose, 0.5% peptone, 0.3% malt extract, and 0.3% yeast extract under aerobic conditions at 37˚C for 12 h at 150 rpm. Gas Chromatography-Mass Spectrometry (GC/MS) analysis confirms the yeast’s efficacy in degrading toxic MG dye complex compounds into simple harmless metabolites. Fourier Transform Infrared (FTIR) analyses show formation of novel compounds post the degradation of MG dye. Further research is advised to identify and characterize the specific enzymes or metabolic pathways implicated in MG dye degradation by *R. mucilaginosa* AUMC13567. Cytotoxicity tests carried out on three human cell lines, including colorectal cancer, head and neck cancer, and healthy skin fibroblast, demonstrating that decolorized MG solution produces safe products devoid of cytotoxicity on tested human cell lines.

**Supplementary Information:**

The online version contains supplementary material available at 10.1186/s12896-025-00977-3.

## Introduction

The rise of chemical synthesis has led to the creation of approximately 10,000 dye substances with diverse colors, contributing to an annual global production of about 7108 tons of dye materials and intermediates [1]. The textile industry alone produces over 7 × 10^7^ tons of synthetic textile dye each year [2, 3]. However, this widespread manufacturing has resulted in ecosystem pollution, affecting air, soil, water, and living organisms near dye manufactories [2]. Industrial textile dye effluents discharged into water bodies alter water characteristics, harming aquatic ecosystems and terrestrial plants. Malachite green (MG), extensively used in various industries, exemplifies the challenges of dye pollution, despite its efficacy and affordability. Malachite green has been associated with severe health risks, impacting various human organs and causing cytotoxicity, genotoxic effects, and skin-related issues [2–5].

Many yeasts have the biodegradation ability of textile dyes in wasted water including *Candida*,* Debaryomyces hansenii*,* Kluyveromyces*,* Pichia occidentalis*,* Rhodotorula*,* Scheffersomyces spartina*,* Sterigmatomyces halophiles*,* Saccharomycopsis lipolytica*,* Saccharomyces uvarum*,* S. cerevisiae*, and *Trichosporon.* Yeasts have high rates of growth and could overcome the hard environmental conditions such as low pH, high potential to absorb, and degradative [2–6, 8–11].

Conventional methods for treating textile industrial effluent wastewater involve expensive technologies, often resulting in incomplete degradation products. In contrast, biological approaches offer a more accessible, cost-effective, and environmentally friendly alternative. Among various microorganisms, yeasts stand out for their rapid growth, tolerance to adverse conditions, and efficient degradation capabilities. *Rhodotorula* sp. exhibits a strong capacity to deal with diverse environmental pressures, including hazardous compounds, and is capable of using various kinds of organic and inorganic substances, giving it an appropriate option for bioremediation applications. Numerous *Rhodotorula* species produce enzymes, including laccases, peroxidases, and other oxidative enzymes, which are capable of degrading complex aromatic compounds. In addition to its ability for rapid growth rates and biomassproduction, which is beneficial for large-scale applications in the bioremediation of dye-contaminated environments [12–17]. reported that *Rhodotorula mucilaginosa*, a red basidiomycetous yeast, has gained attention for its biotechnological significance, producing carotenoids, lipids, enzymes, and bioactive metabolites [18]. recorded that *Rhodotorula* sp. MZ312369 was good candidate for bioremediation of heavy metals such as Zn and Cr due to its ability to produce carbonic anhydrase enzyme (CA).

This study considered as the first study correlated between MG dye bioremediation and *R. mucilaginosa* yeast strain. We evaluated the environmental and nutritional factors for raising MG dye decolourization by tested yeast strain *R. mucilaginosa* AUMC13567. Also, this work identifies the MG biodegradation metabolites by GC/MS and FTIR Analysis, in addition **to** determine their cytotoxicity capacity via the human cell lines including colorectal cancer, head and neck cancer, and healthy skin fibroblast.

## Materials and methods

### Yeast strain and culture conditions

The tested strain *Rhodotorula mucilaginosa* AUMC13567 was isolated from molasses in previous study in our laboratory [16] and grown on yeast extract malt extract peptone glucose medium (YME) sterilized solid media composed of (Distilled water, 2% agar, 0.5% peptone, 0.3% yeast extract, 0.3% malt extract, 1% glucose). The yeast inoculum prepared by pre-cultured in 250 mL conical flask containing 50mL of sterilized broth medium at 28 ± 2ºC in shaking incubator at 100 rpm for 72 h [19, 20]. For testing the ability of *R. mucilaginosa* AUMC13567 for biodegradation of malachite green dye, it cultivated at rate 10% in 250 mL conical flask included 50 mL sterilized YME broth medium and 50 mg/L of malachite green dye in 3 replicates. The pH was then adjusted to 5.8 flasks were incubated in shaking incubator at 100 rpm for 3 days at 28 ± 2^o^C.

### Decolorization bioassay

The decolorization assay according to [21–28] described as following: A five ml sample was separated from the cell mass by centrifugation at 5000 × g for 7 min. Under cold conditions (CRU-5000 Centrifuge IEC) and three ml of supernatant was withdrawn, and measured at (620 nm.). The detection was made in triplicate using Mnicam/UV-vis spectrophotometry Helios Gamma, at the Botany and Microbiology Department, Faculty of Science, Assiut University.

The percentage decolorization was calculated as follows

$$\begin{aligned}&\text{D}\text{e}\text{c}\text{o}\text{l}\text{o}\text{r}\text{i}\text{z}\text{a}\text{t}\text{i}\text{o}\text{n}\:\text{p}\text{e}\text{r}\text{c}\text{e}\text{n}\text{t}\text{a}\text{g}\text{e}\:\cr&=\frac{\text{I}\text{n}\text{t}\text{i}\text{a}\text{l}\:\text{a}\text{b}\text{s}\text{o}\text{r}\text{b}\text{a}\text{n}\text{c}\text{e}-\text{O}\text{b}\text{s}\text{e}\text{r}\text{v}\text{e}\text{d}\:\text{a}\text{b}\text{s}\text{o}\text{r}\text{b}\text{a}\text{n}\text{c}\text{e}\:}{\text{I}\text{n}\text{i}\text{t}\text{i}\text{a}\text{l}\:\text{a}\text{b}\text{s}\text{o}\text{r}\text{b}\text{a}\text{n}\text{c}\text{e}}\text{*}100\end{aligned}$$

### Optimization of culture conditions for maximum degradation efficiency

#### Effect of three types of growth media

Malachite green concentration was (50 mg/L) used in each three types of medium tested which were:

- Plain distilled water.
- Distilled water with 5% glucose.
- modified YME medium which composed of (Distilled water, 3% glucose, 0.5% peptone, 0.3% malt extract and 0.3% yeast extract). Flasks were incubated at 28 °C, under shaking (100 rpm) for 72 h. And the percent of color removal was calculated as indicated above, the color of yeast cells was examined under light microscope after 72 h.

### Effect of incubation type

Two different types of cultivation were studied included submerged cultivation (shaking culture) was carried out in an incubator shaker (Environ-Shaker 3597-1) at 100 rpm and static cultivation (surface culture) was performed on static incubator. In each type we used 250 ml flasks contained 50mL sterilized modified YME media with 50 mg/L of MG dye concentration then they incubated at 28 °C for 72 h. And the percent of color removal was calculated as indicated above, the color of yeast cells was examined under light microscope after 72 h.

### Effect of different agitation speed

Five different agitation speeds were tested (50, 80, 100, 120 and 150) rpm by cultivation the flasks in incubator shaker. Flasks were incubated under the previous conditions. And the percent of color removal was calculated as indicated above, the color of yeast cells was examined under light microscope after 72 h.

### Effect of different incubation temperature

Effect of four different temperature degrees on MG dye decolourization was tested: (25, 28, 30, and 37 °C). Flasks were incubated on modified YME medium with 50 mg/L MG dye concentration under shaking (150 rpm) for 72 h. And the percent of color removal was calculated as indicated above, the color of yeast cells was examined under light microscope after 72 h.

### Effect of different malachite green concentrations

Effect of different malachite green concentrations (50, 100, 200, 300, 500 and 1000 mg/L) on the decolourization efficiency by ***R. mucilaginosa*** was studied. The flasks were prepared as described previously, incubated at 37 °C under shaking condition (150 rpm) for 72 h. The color removal was calculated as indicated above; the color of yeast cells was examined under light microscope after 72 h.

### Statistical analysis

All tests were performed in triplicate. Data with a non-normal distribution were expressed as the mean ± Standard Error (SE) of at least three independent experiments. Significant differences among means were identified using the ANOVA test followed by Duncan ^a^ post hock test. The data were analyzed using IBM SPSS Statistics 21. Values of *p* < 0.05 were considered statistically significant.

### GC/MS and FTIR analysis

To confirm biodegradation of MG textile dye and to identify produced metabolites we used GC/MS device Model of GC-MS Apparatus (7890 A-5975B) and Column (DB-5ms), the metabolites resulted from the biodegradation of malachite green at zero time “control” and 12 h “after decolorization of dye solution” were extracted with equal volumes of ethyl acetate; dried the extract over Na_2_SO_4_ anhydrous and evaporated in a rotary evaporator to dryness. Derided crystals were dissolved in a small volume of pure methanol for GC/MS analysis under the following conditions: Oven program, 40 °C for 2 min; then 10 °C/min to 150 °C for 3 min; 10 °C/min to 220 °C for 6 min; then 15 °C/min to 280 °C for 28 min. Run Time, 61 min and 2 min (Post Run) 260 °C. Flow program, 0.5 mL/min for 10.9 min; then 1 mL/min per min to 1 mL/min for 30 min.

The same sample was used for FTIR analysis that carried out using a Perkin-Elmer spectrophotometer at 783 and changes in percentage transmission at different wavelengths were observed.

### Effect of MG biodegradable metabolites on cell line tests

#### Cell culture

Three kinds of cell lines were used for bioassay includes Colorectal Cancer (CaCo-2), Head and neck cancer (Hep), and Human Skin Fibroblast (HSF), all cell-based experiments were performed at Nawah Scientific Research Laboratory (Mokatam, Cairo, Egypt). The cell lines used in this study were provided by Nawah Scientific, which maintains authenticated and validated cell lines as part of its research services. The cells were cultured according to Nawah’s standard protocols, ensuring proper cell line identity and viability throughout the experiments. CaCo-2 Cells were maintained in RPMI media, while Hep-2 and HSF cells were maintained in DMEM media. Both media were supplemented with 100 mg/mL of streptomycin, 100 units/mL of penicillin and 10% of heat-inactivated fetal bovine serum in humidified, 5% (v/v) atmospheric CO_2_ at 37 °C.

#### Cytotoxicity assay

By SRB assay, the cell viability was assessed. Aliquots of 100 µL cell suspensions (5 × 10^3^ cells) were in 96-well plates and incubated in complete media for 24 h. Cells were treated with 100 µL media containing drugs at various concentrations, after 72 h of drug exposure, cells were fixed by replacing media with 150 µL of 10% TCA and incubated at 4 °C for 1 h, then removing TCA solution, and washing cells 5 times with distilled water. Aliquots of 70 µL SRB solution (0.4% w/v) were added and incubated in a dark place at room temperature for 10 min. Plates washed 3 times with 1% acetic acid and allowed to air-dry overnight. Then, 150 µL of TRIS (10 mM) was added to dissolve protein-bound SRB stain; the absorbance was measured at 540 nm using a BMG LABTECH^®^- FLUO star omega microplate reader “Ortenberg, Germany” [29].

## Results

Tables 1_a − e_ showed the enhancement of the decolorization capacity of *R. mucilaginosa* AUMC13567, various parameters encompassing nutritional and environmental factors, were manipulated to optimize decolorization.

**Table 1:** Optimization parameters for decolorize the MG textile dye by *R. mucilaginosa* **AUMC13567**

a] Effect of three types of growth media				b] Type of Incubation		c] Agitation speed						
Incubation by hours	a)Plain water	b) Glucose	c) Media	Static	Submerged	50rpm	80rpm	100rpm	120rpm	150rpm		F-value
**2hrs**	3.8 ± 0.1^a1^	12.3 ± 0.2 ^b1^	15.3 ± 0.2 ^c1^	7.7 ± 0.2^1^	15.3 ± 0.2 ^1^	4.8 ± 0.5^a1^	5.9 ± 0.1 ^b1^	26.2 ± 0.1 ^c1^	42.9 ± 0.4 ^d1^	60.7 ± 0.0^e1^		7050^***^
**4hrs**	5.6 ± 0.6^a1^	14.1 ± 0.2 ^b1^	26.0 ± 0.4 ^c2^	11.7 ± 0.3^2^	26.0 ± 0.4 ^2^	7.8 ± 0.2^a2^	15.0 ± 5.9 ^a2^	27.6 ± 0.2 ^b1^	49.4 ± 0.7^c2^	80.4 ± 0.1^d2^		120^***^
**6hrs**	10.8 ± 0.3^a2^	21.8 ± 1.9 ^b2^	55.9 ± 0.2^c3^	45.9 ± 0.1^3^	55.9 ± 0.2 ^3^	11.0 ± 0.8^a3^	11.2 ± 1.3 ^a3^	61.0 ± 0.1 ^b2^	54.2 ± 0.2^c3^	96.4 ± 0.1 ^d3^		2669^***^
**8hrs**	20.4 ± 2.0^a3^	37.5 ± 0.6 ^b3^	75.5 ± 1.0 ^c4^	51.1 ± 1.1^4^	75.5 ± 1.0 ^4^	16.7 ± 0.2^a4^	16.7 ± 0.3 ^a4^	68.9 ± 1.0 ^b3^	57.0 ± 0.8^c4^	97.8 ± 0.0 ^d4^		3717^***^
**10hrs**	41.8 ± 0.1^a4^	50.5 ± 0.1^1b4^	87.0 ± 0.3 ^c5^	54.1 ± 1.0^5^	87.0 ± 0.3 ^5^	25.4 ± 0.2^a5^	27.1 ± 0.3^b5^	87.4 ± 0.4 ^c4^	67.2 ± 0.1^d5^	99.5 ± 0.0 ^e5^		26661^***^
**12hrs**	**70.7 ± 0.6** ^**a5**^	**83.3 ± 0.1** ^**b5**^	**92.0 ± 0.0** ^**c6**^	56.0 ± 0.2^6^	**92.0 ± 0.0** ^**6**^	29.2 ± 0.1^a6^	32.0 ± 0.1 ^a6^	94.1 ± 1.2 ^b5^	95.8 ± 1.9^b6^	100 ± 0.0 ^c6^		1310^***^
**F-value**	854***	4568^***^	1091^***^	1238	4568	437^***^	312^***^	2503^***^	442^***^	97832^***^		
Completely decolorize	56hr	32hr	24hr	50 hrs.	24hr	76h	72h	30 h	24 h			
**d] Incubation temperature degree**						**e] Malachite Green Dye Concentrations**						
**Incubation by hours**	**25˚c**	**28 ˚c**	**30 ˚c**	**37 ˚c**	**F-value**	**50mg**	**100mg**	**200mg**	**300mg**	**500mg**	**1000mg**	**F-value**
**2hrs**	7.0 ± 0.1^a1^	15.3 ± 0.2 ^b1^	26.2 ± 0.1 ^c1^	60.7 ± 0.0 ^d1^	28006^***^	60.7 ± 0.0^e1^	54.2 ± 0.9 ^d1^	4.5 ± 0.0 ^b1^	9.6 ± 0.0 ^c1^	0.2 ± 0.1^a1^	0.0 ± 0.0 ^a^	5931^***^
**4hrs**	12.4 ± 0.1^a2^	26.0 ± 0.4 ^b2^	27.6 ± 0.2 ^c1^	80.4 ± 0.1 ^d2^	15418^***^	80.4 ± 0.1^d2^	81.6 ± 0.1 ^e2^	12.8 ± 0.0 ^c2^	13.0 ± 0.1^c2^	1.9 ± 0.0^b2^	0.0 ± 0.0^e^	299943^***^
**6hrs**	52.5 ± 0.1^a3^	52.6 ± 0.4 ^a3^	61.0 ± 0.1 ^b2^	96.4 ± 0.1 ^c3^	12466^***^	96.4 ± 0.1^f3^	92.2 ± 0.2 ^e3^	15.8 ± 0.0^d3^	13.7 ± 0.0^c3^	3.5 ± 0.0 ^b3^	0.0 ± 0.0^a^	156052^***^
**8hrs**	56.9 ± 0.1^a4^	62.3 ± 0.8 ^b4^	68.9 ± 1.0 ^c3^	97.8 ± 0.0 ^d4^	833***	97.8 ± 0.0^e4^	97.9 ± 0.1^e4^	18.3 ± 0.1 ^d4^	14.4 ± 0.0^c4^	3.5 ± 0.0 ^b3^	0.0 ± 0.0 ^e^	541877^***^
**10hrs**	59.1 ± 0.0^a5^	87.0 ± 0.3 ^b5^	87.4 ± 0.4 ^b4^	99.5 ± 0.0 ^c5^	4225***	99.5 ± 0.0^e5^	99.6 ± 0.0^e5^	28.2 ± 0.7 ^d5^	14.4 ± 0.1^c4^	3.5 ± 0.0 ^b3^	0.0 ± 0.0 ^a^	27423^***^
**12hrs**	66.5 ± 0.3^a6^	92.0 ± 0.0 ^b6^	94.1 ± 1.2 ^c5^	**100 ± 0.0** ^**d6**^	575***	**100 ± 0.0** ^**e6**^	**99.7 ± 0.0** ^**e5**^	32.5 ± 1.0 ^d6^	14.4 ± 0.1^c4^	3.5 ± 0.0^b3^	0.0 ± 0.0 ^a^	12754^***^
**F-value**	35472^***^	5096***	1952^***^	97832***		97832^**^	2184^***^	418^***^	663^***^	2413^***^		
Completely decolorize	72hr	30hr	24hr				After 13hrs	After 72hrs				

All results are compared to each other at *P* < 0.05. Values with the different superscripts along the same column are statistically different from each other ***p < 0.001. by One Way Anova values comparison. Post Hoc Tests: Duncan a *p* < 0.05 is represented by superscripts 1–10 and different superscripts along the same row are statistically different from each other ***p < 0.001. by One Way Anova values comparison. Post Hoc Tests: Duncan a *p* < 0.05 is represented by superscripts a-f

### Effect of different types of media

Table 1_a_ shows the maximum decolorization percentages after 12 h for (a) plain distilled water was (70.7 ± 0.6%), (b) distilled water with 5% glucose was (83.3 ± 0.1%), and (c) modified YME medium was (92 ± 0.0), complete removal of MG dye color occurred on medium (c) after 24 h, on medium (b) after 32 h and on medium (a) after 56 h.

#### Effect of incubation type

Table 1_b_ shows the decolorization efficiency of MG dye by R. *mucilaginosa* under shaking and static cultivation, indicating significantly higher decolorization percentage under shaking was (92.0 ± 0.0%) compared to static cultivations was (56.0 ± 0.2%). Complete decolorization occurred after 24 h under shaking conditions and after 50 h when cultivation was static.

#### Effect of agitation speed

Increasing agitation speed, as shown in table 1_c_, enhanced the ability of yeast strain for MG decolorization. 100% decolorization was achieved at 150 rpm after 24 h.

#### Effect of incubation temperatures

Table 1_d_ demonstrates that higher incubation temperatures and longer times resulted in increased decolorization percentages, with 100% achieved at 37 °C after 12 h.

#### Effect of MG dye concentrations

Table 1_e_ shows the impact of MG concentrations on the yeast strain’s decolorization ability. Complete decolorization occurred at 50 mg/L, with higher concentrations exhibiting reduced decolorization efficiency. And at MG dye concentration 100 mg/L the decolorization percentage was 99.7% after 12 h.

The results of yeast cells examination under light microscope at maximum decolorization percentage 100% showed that the yeast cells were colorless and there were no MG dye particles was precipitated inside or outside the yeast cells. Which indicated that the ability of yeast to decolorize the color of the dye is due to its ability to degrade the molecules of MG dye.

#### MG dye biodegradation pathway

**Fig. 1:**
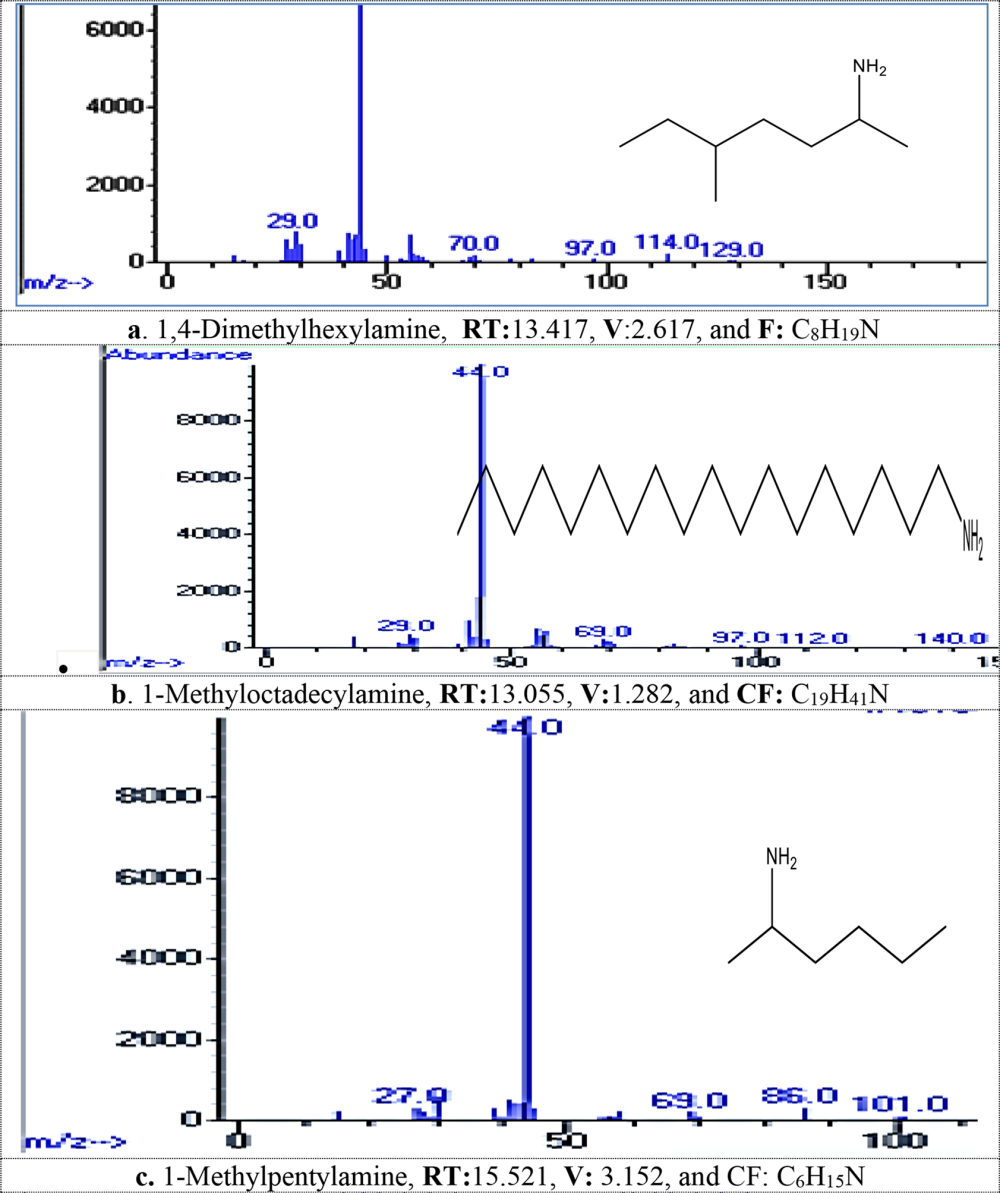

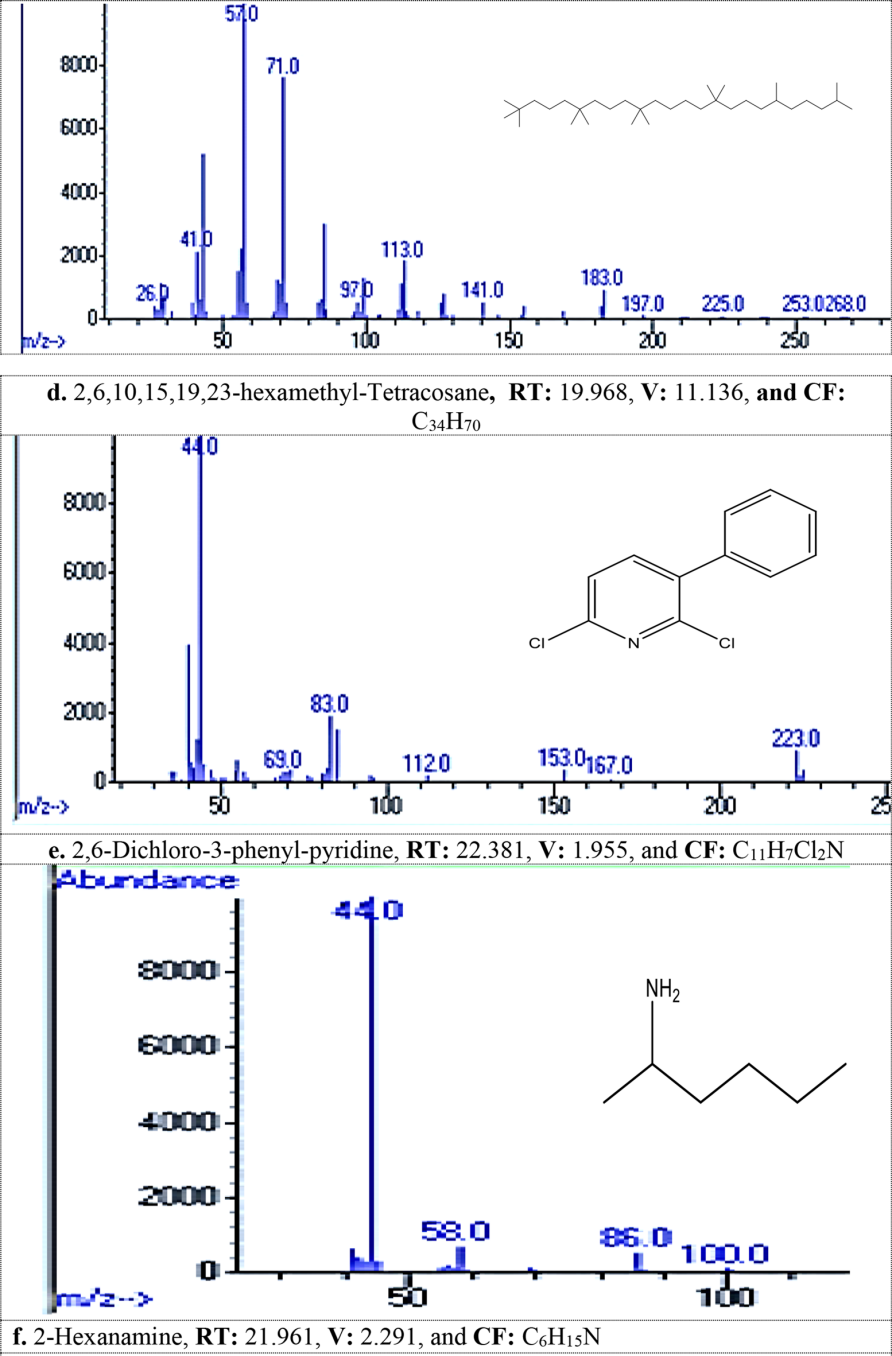

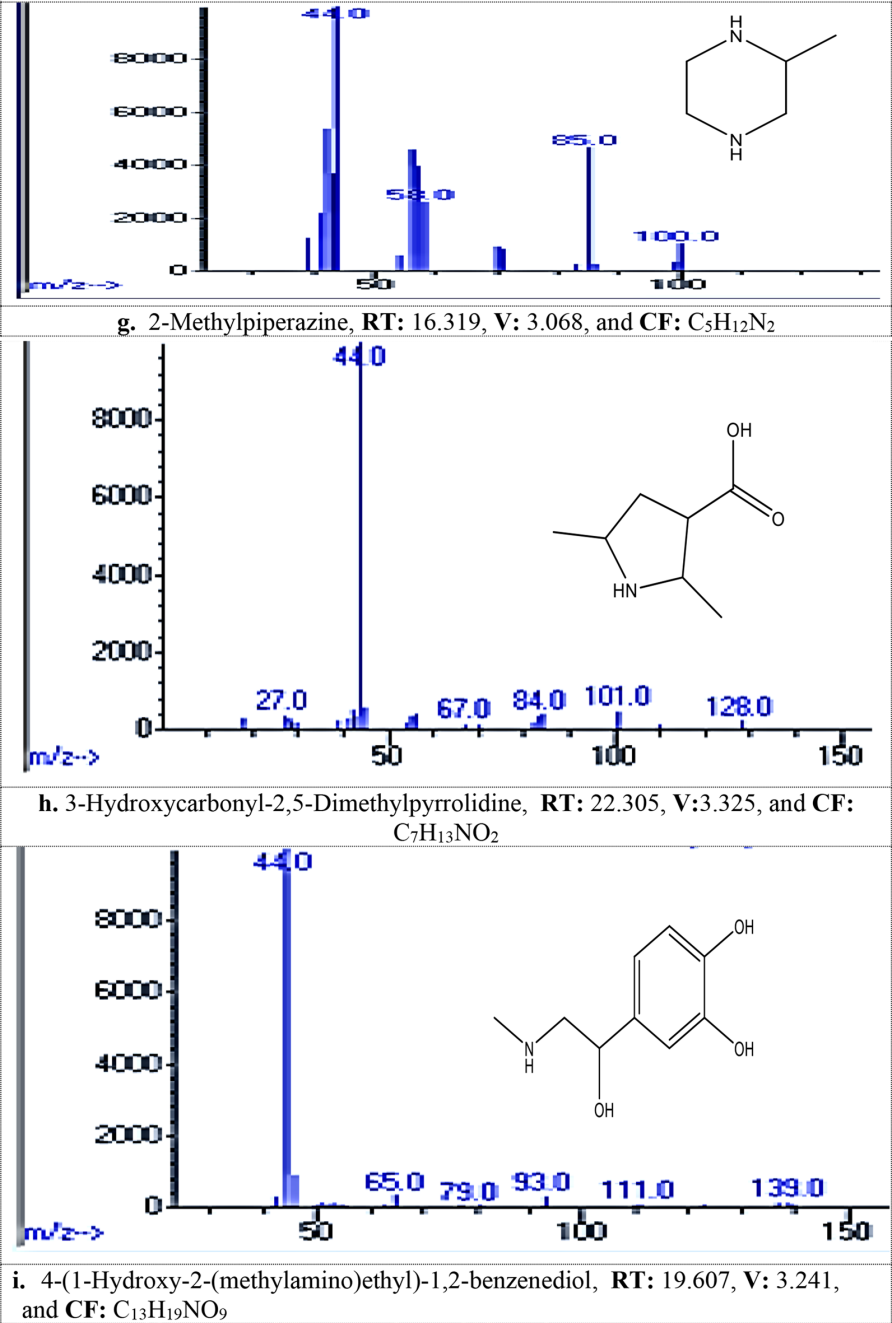

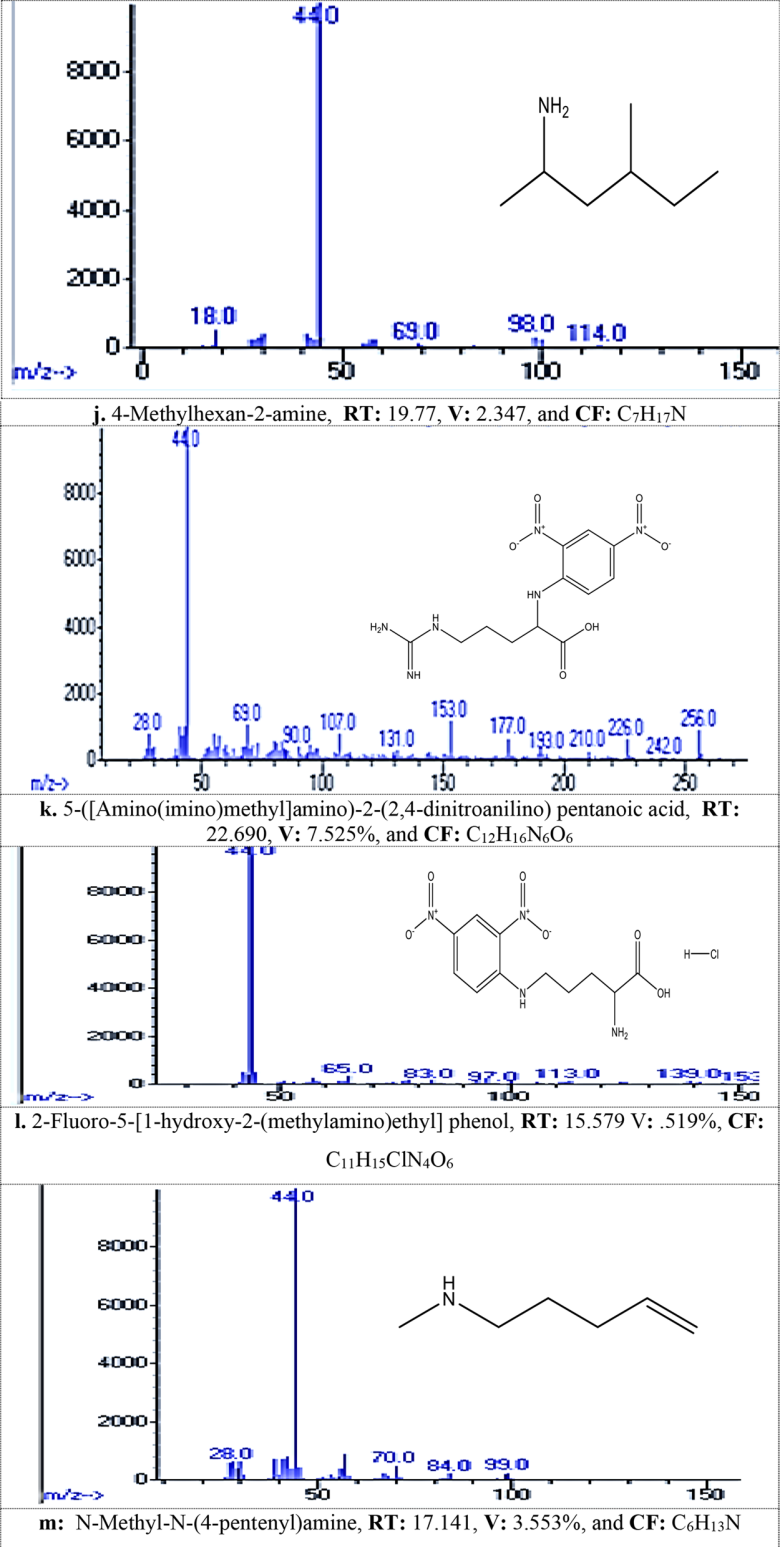
_**a**− **m**_: The spectra of metabolites after MG degadative by tested *R. mucilaginosa* AUMC13567 detected by GC/MS analysis (**a**-**m**): Various metabolite, RT: Retention Time min, V: Value, and CF: Chemical Formula

**Fig. 2:**
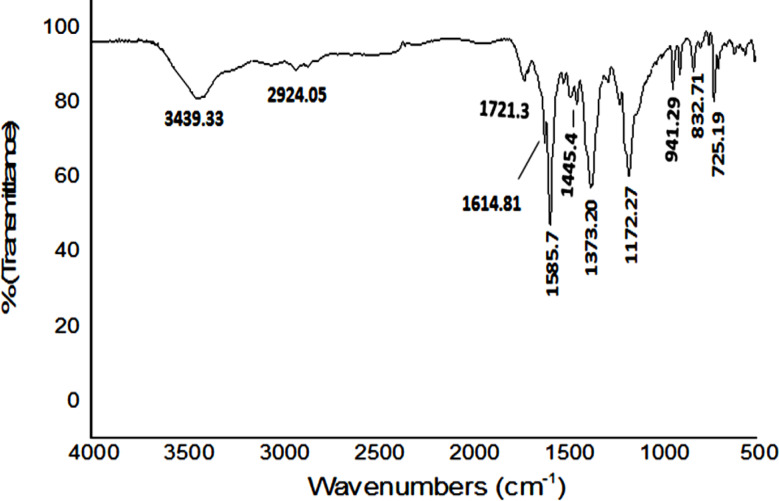
FTIR spectrum of MG dye before degradation at 0 h

**Fig. 3:**
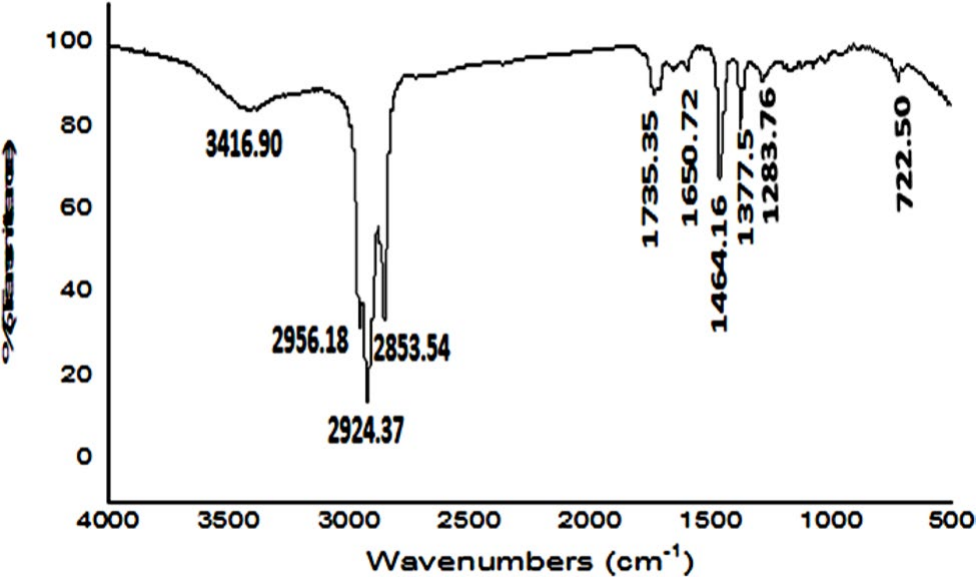
FTIR spectrum of MG metabolites produced by cultivation Rhodotorula *mucilaginosa* AUMC13567 after 12 h cultivation period

GC/MS analysis detected 13 metabolites resulted from biodegradation of MG dye, Table 2; Fig. 1_**a** − **m**_. The produced metabolites including different compounds containing easily biodegradable functional groups of the ring substituents such as phenolic, alcohol, amino, or other that produced from aromatic cleavage. FTIR analysis Table 3; Figs. 2 and 3 confirmed the biodegradation of MG dye to different products with different peaks in comparing to undegraded MG dye representing carboxylic acid, alkenes, alcohol, and halo compounds.

**Table 2:** Metabolites detected by gc/mass generated during malachite green dye degradation by *R. mucilaginosa* AUMC13567 yeast strain

Name	Retention Time (min)	Chemical Formula
1,4-Dimethylhexylamine	13.417	C_8_H_19_N
1-Methyloctadecylamine	13.055	C_19_H_41_N
1-Methylpentylamine	15.521	C_6_H_15_N
2,6,10,15,19,23-hexamethyl-Tetracosane	19.968	C_34_H_70_
2,6-Dichloro-3-phenyl-pyridine	22.381	C_11_H_7_Cl_2_N
2-Hexanamine	21.961	C_6_H_15_N
2-Methylpiperazine	16.319	C_5_H_12_N_2_
3-Hydroxycarbonyl-2,5-Dimethylpyrrolidine	22.31	C_7_H_13_NO_2_
4-(1-Hydroxy-2-(methylamino)ethyl)-1,2-benzenediol	19.607	C_13_H_19_NO_9_
4-Methylhexan-2-amine	19.77	C_7_H_17_N
5- Amino(imino)methyl]amino)-2-(2,4-dinitroanilino) pentanoic acid	22.690	C_12_H_16_N_6_O_6_,
2-Fluoro-5-[1-hydroxy-2-(methylamino)ethyl] phenol,	15.579	C_11_H_15_ClN_4_O_6_
N-Methyl-N-(4-pentenyl)amine	17.141	C_6_H_13_N

**Table 3:** FTIR spectrum analysis of most important functional groups in malachite green dye solution before and after degradation by *R. mucilaginosa* AUMC13567 yeast strain

Malachite green dye solution (before degradation at 0 h)		Malachite green dye solution (after yeast degradation at 12 h)	
Wave length (cm^− 1^)	Possible Functional Group(s)	Wave length (cm^− 1^)	Possible Functional Group(s)
3439.33	–OH or –NH stretch (hydroxyl or amine)	3416.90	O–H stretch (hydrogen-bonded hydroxyl groups)
2924.05	C–H stretch (alkane or aromatic C–H)	2956.18	C–H stretch (alkyl groups)
1721.3	C = O stretch (carbonyl group)	2924.37	C–H stretch (alkyl groups)
1614.81	C = C stretch (aromatic ring), or C = N stretch	2853.54	C–H stretch (alkyl groups)
1585.7	Aromatic C = C stretch or N–H bend	1735.35	C = O stretch (carbonyl group)
1445.4	C–H bending (CH₂ or CH₃)	1650.72	C = C stretch (conjugated alkenes)
1373.20	CH₃ symmetric bending	1464.16	C–H bending (methylene groups)
1172.27	C–N or C–O stretch	1377.5	C–H bending (methyl groups)
941.29	C–H out-of-plane bending (aromatic ring)	1283.76	C–O stretch (ethers, esters)
832.71	C–H out-of-plane bending (aromatic)	722.50	C–H out-of-plane bending (aromatic rings)
725.19	C–H bending (possibly –(CH₂)_n_ – rocking)	-	-

**Fig. 4:**
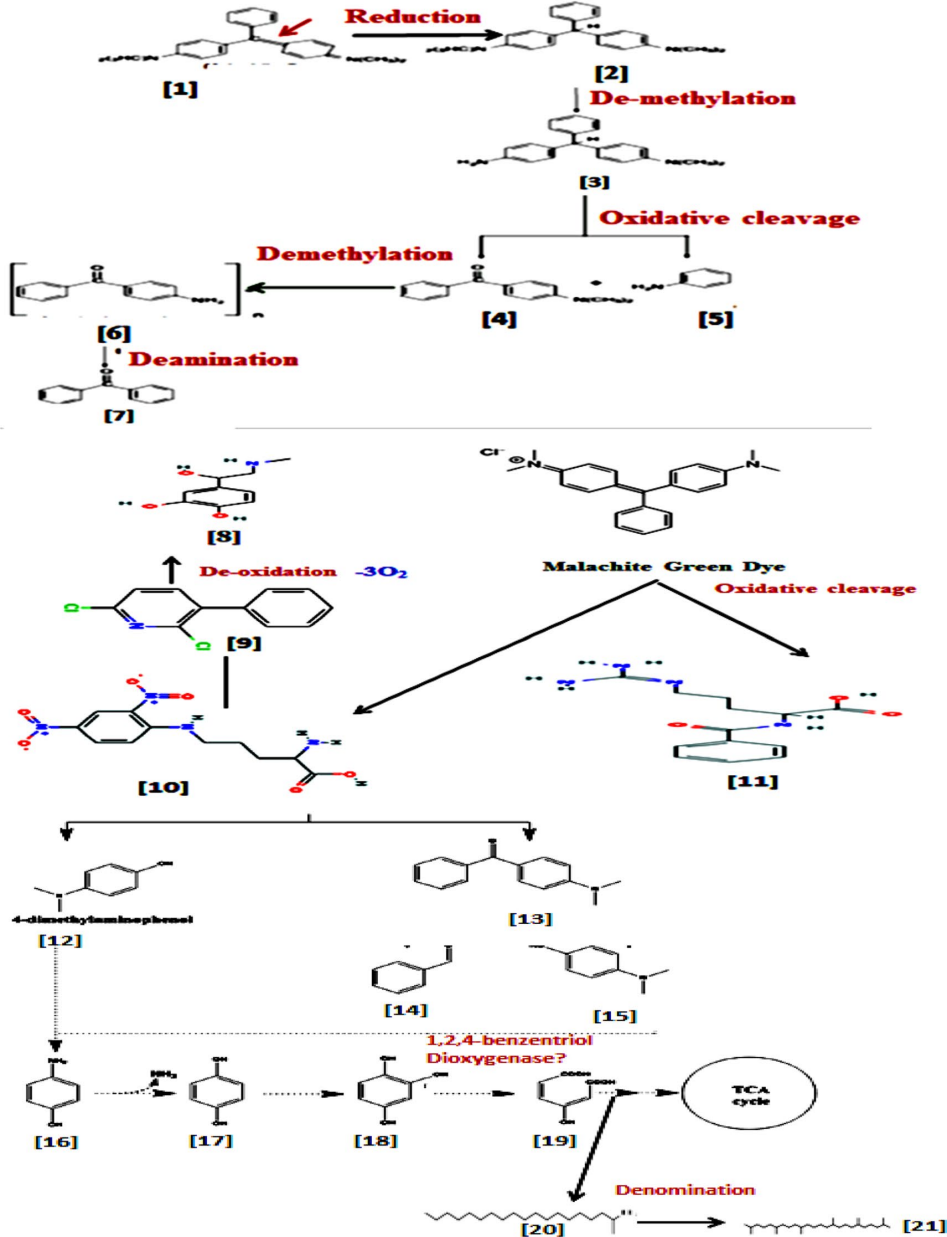
Proposed mechanism of decolorization and degradation of MG by *R. mucilaginosa* AUMC13567 includes oxidative clave, reductase enzyme, demethylation, oxidative clave, demethylation, and deamination [1]. Malachite Green Textile Dye= [4-{[4-(Dimethyl amino) phenyl](phenyl) methyldiene}-N, N-dimethyl cyclohexa-2,5-dien-1-iminium chloride] **(C**_**23**_**H**_**25**_**ClN**_**2**_**).** [2] Leuco-malachite green dye without 2 double bonds [3]. 4-{(4-aminophenyl) (phenyl)methyl] N-N-dimethyl [4]. 4-dimethylaminobenzophenone [5]. Aniline [6]. 4-aminobenzophenone [7]. Benzophenone [8]. 4-(1-Hydroxy-2-(methylamino) ethyl)-1,2-benzenediol **(C**_**13**_**H**_**19**_**NO**_**9**_**).** [9] 2,6-Dichloro-3-phenyl pyridine (C11H7Cl2N) [10]. 2-Amino-5-(2,4-dinitroanilino) pentanoic acid; hydrochloride **(C**_**11**_**H**_**15**_**ClN**_**4**_**O**_**6**_**).** [11] 5-([Amino(imino)methyl] amino)-2-(2,4-dinitroanilino) pentanoic acid **(C**_**13**_**H**_**18**_**N**_**4**_**O**_**3**_**).** [12] 4-dimethylaminophenol [13]. 4-(dimethyl-aminophenol)-benzo-phenone [14]. Benz-aldehyde [15]. 4-(dimethyl-aminophenol) [16]. 4-aminophenol [17]. 1,4 benzendiol [18]. 1,2,4 benzentriol [19]. Maleyl-acetic acid [20]. 1-Methyloctadecylamine(2-Nonadecanamine) **(C**_**19**_**H**_**41**_**N).** [21] 2,6,10,15,19,23-Hexamethyl-2,6,10,15-tetramethyl-tetracosane (**C**_**34**_**H**_**70**_**)**

Figure 4, discusses the proposed mechanism of biodegradation of MG dye by *R. mucilaginosa* AUMC13567 includes oxidative clave, reductase enzyme, demethylation, oxidative clave, demethylation, and deamination.

#### Effect on three human cell lines

**Fig. 5:**
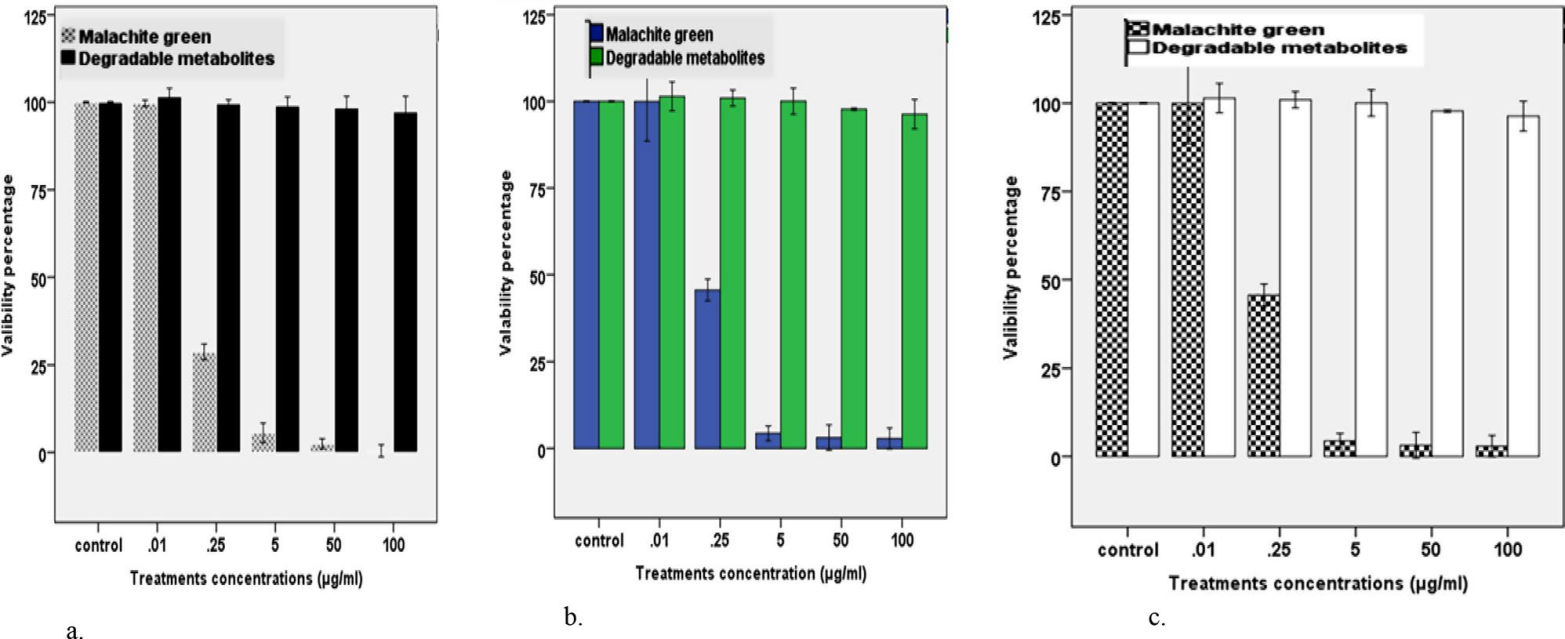
_**a**− **c**_: Concentration and time-dependent response curves for MG and degradable metabolites tested in three cells lines. Results are expressed as mean ± SE of three independent experiments (**a**) Human Skin Fibroblast (HSF) (**b**) Head and neck cancer-2 (Hep) (**c**) Colorectal cancer-2 (Coca)

**Fig. 6:**
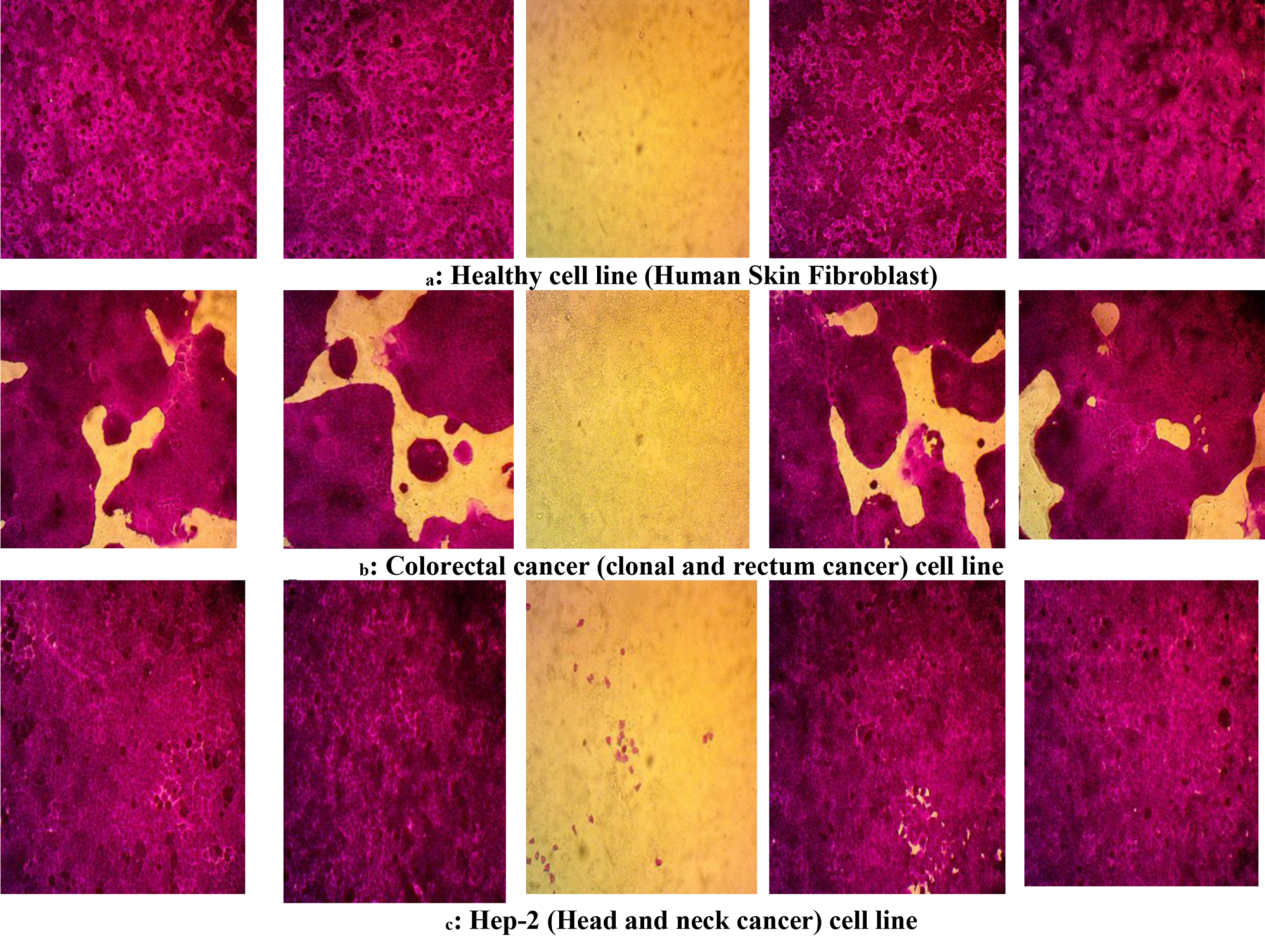
_**a**−**c**_: Morphological changes on three types of human cell lines treated by MG and degradable metabolites were MG: Malachite green, **BMGM**: Biodegradable Malachite Green Metabolites by yeast

Cytotoxicity assays on three human cell lines (CaCo-2, Hep-2, HSF) exposed to MG and its degradable metabolites, showing that the MG textile dye has high toxicity and their biodegradable metabolites recorded minimal cytotoxicity Table 4; Figs. 5_a − c_ & 6_a−c_.

**Table 4:** Cytotoxicity of MG and MG decolorized by *R. mucilaginosa* AUMC13567 on three cell lines

Cell line	Hep-2		HSF		Coca	
Tested compounds	MG	DM	MG	DM	MG	DM
Blank	0.069	0.074	0.082	0.090	0.082	0.079
Control	5.28 ± 0.03^c^	5.37 ± 0.04^b, c^	5.43 ± 0.07^d^	5.43 ± 0.07^d^	4.64 ± 0.05 ^c^	4.62 ± 0.05
0.01	5.28 ± 0.14^c^	5.44 ± 0.05^c^	5.41 ± 0.01^d^	5.41 ± 0.01^d^	4.67 ± 0.02 ^c^	4.57 ± 0.07
0.25	2.41 ± 0.04^b^	5.42 ± 0.03^c^	1.56 ± 0.03^c^	1.56 ± 0.03^c^	1.89 ± 0.07 ^b^	4.55 ± 0.01
5	0.23 ± 0.03^a^	5.37 ± 0.05^b, c^	0.30 ± 0.04^b^	0.30 ± 0.04^b^	0.05 ± 0.01 ^a^	4.51 ± 0.05
50	0.16 ± 0.05^a^	5.25 ± 0.0^a, b^	0.14 ± 0.02^a^	0.14 ± 0.02^a^	0.03 ± 0.02 ^a^	4.45 ± 0.08
100	0.15 ± 0.04^a^	5.17 ± 0.05^a^	0.03 ± 0.02^a^	0.03 ± 0.02^a^	0.02 ± 0.00 ^a^	4.44 ± 0.05
F-value	1444 ***	7***	5062***	5062***	3872***	1

Values are expressed as means ± SEM (Std. Error of Mean). Values with different superscript letters in the same column for each parameter are significantly different from each other at (*P* < 0.05), cell line represented as: Coca (clonal and rectum cancer), Hep-2 (Head and neck cancer), healthy HSE cell line (Human Skin Fibroblast) and the terminated group MG: malachite green, DM: Decolorized metabolites

Morphological changes in the colorectal cancer (CaCo-2) cell line were examined after being treated by a control sample, Malachite Green, and Malachite Green after degradation by *R. mucilaginosa* AUMC13567. The results show a difference between the CaCo-2 cells after being treated by control they had a regular shape and there is viability noted in cells that appeared colored, with the cells that lost viability as they lost their regular shape and appeared colorless after incubation with Malachite Green for 48 h especially at 100 µg mL^− 1^, about %100 of cells disappear may indicate its death. There is no difference in the morphological shape of the CaCo-2 cell after being treated by control and the morphological shape of the CaCo-2 cell after being treated by Malachite Green at 0.01 µg mL^− 1^. This indicate the slight effect of Malachite Green at this concentration. Also, there is no difference in the morphological shape of the CaCo-2 cell after being treated by control and the morphological shape of the CaCo-2 cell after being treated by both concentration (0.01 and 100 µg/mL^− 1^) of Malachite Green degradation by *R. mucilaginosa*_AUMC13567_ the cells remind regular in shape and viability noted in cells which appeared colored.

There is no difference in the morphological shape of the Head and neck cancer cells after being treated by control and the morphological shape of the Head and neck cancer cells after being treated by Malachite Green at 0.01 µg/mL^− 1^. Also, there is no difference in the morphological shape of the Head and neck cancer cells cell after being treated by control and the morphological shape of the Head and neck cancer cells after being treated by both concentration (0.01 and 100 µg/mL^− 1^) of Malachite Green degradation by *Rhodotorula mucilaginosa* AUMC13567 the cells remind regular in shape and viability noted in cells which appeared colored.

Morphological changes in the Human Skin Fibroblast (HSF) cell line were examined after being treated by a control sample, Malachite Green, and Malachite Green after degradation by *Rhodotorula mucilaginosa* AUMC13567.

The results show a difference between the Human Skin Fibroblast (HSF) cells after being treated by the control they had a regular shape and there is viability noted in cells that appeared colored, with the cells that lost their regular shape and seemed to be colorless after incubation with Malachite Green for 48 h especially at 100 µg/mL^− 1^. About 100% of cells disappear, which may indicate their death.

There is no difference in the morphological shape of the Human Skin Fibroblast (HSF) after being treated by control and the morphological shape of the Human Skin Fibroblast (HSF) after being treated by Malachite Green at 0.01 µg/mL^− 1^. Also, there is no difference in the morphological shape of the Human Skin Fibroblast (HSF) cell after being treated by control and the morphological shape of the Human Skin Fibroblast (HSF) after being treated by both concentration (0.01 and 100 µg/mL^− 1^) of Malachite Green degradation by *Rhodotorula mucilaginosa* AUMC13567 the cells remind regular in shape and viability noted in cells which appeared colored.

## Discussion

For the first time the *R. mucilaginous* AUMC13567 is used for biodegradation of MG dye solution in our laboratory. In previous work in our laboratory [16] we examine this strain and revealed that this yeast produces flavonoids (quercetin 0.005) and phenols (gallic 2.6), which are considered as good antioxidant bioactive compounds [30] record that *R. mucilaginosa* is very unique yeast strain in dye bioremediation due to it could produce many important enzymes such as lipase, protease, manganese dependent peroxidase (MnP) and lignin peroxidase (LiP), respectively. In this study three types of medium (distilled water, distilled water with 5% glucose medium, modified YME medium) were used to investigate the ability of *R. mucilaginous* AUMC13567 to decolorize MG dye. Modified YME medium with 3% glucose was the best one of them, may be due to presence of high concentration of carbon (3% glucose) and different sources of nitrogen (peptone and yeast extract) which support high rate of yeast growth, in addition to production of high amount of energy and enzymes which help in MG dye biodegradation process. *Saccharomyces cerevisiae* MTCC463 decolorization percentage of MG textile dye in plain distilled water was 85% after 7 h and about 95.5% in 5% glucose medium within 4 h, under aerobic conditions and at room temperature [26].

During the current work two types of cultivation were studied, where the percentage of MG dye decolorization was much higher under the shaking condition compared to the static one in all incubation period. The complete decolorization 100% of MG dye achieved after 24hs in case of cultivation under shaking and in case of static cultivation it was after 50 h. This is in agreement with the work of [31] which reported that the shaking condition for cultivation was the best and the decolorization percentage of MG dye by *Bacillus cereus* and *Pseudomonas earoginosa* were 92.4 ± 0.9, 95.7 ± 0.4%, respectively. Although, the yeast cell growth was maximized under the shaking condition due to the good distribution of nutrients during shaking [11, 32] recorded that the maximum decolorization of malachite green by *S. pulverulentum*, was 93.3% under agitated conditions, pH of 4.5 and use of glucose as a carbon source. On the other hand, low efficiency of MG decolorization under the static condition in our study was detected, which might be due to the settling of the yeast cells to the bottom of the flasks under this condition and become nutrients and oxygen-depleted that would lead to low rate of yeast cell growth and by sequence low enzymes activity which reduce dye decolorization process as recorded by [33].

In our study the complete decolorization of MG dye by *R. mucilaginosa* tested strain occurs under shaking cultivation condition at high shaking speed 150 rpm. Which could be explained due to increase in agitation speed increases the amount of dissolved oxygen and enhances the oxidative exoenzymes for oxidation and biodegradation of the MG dye.

During study the decolorization percentage of MG dye at different cultivation temperature by *R. mucilaginosa* the complete decolorization 100% was carried out at 37 °C after 12 h which may be due to an increase in thermo-stable biodegradable enzymes. Also [31], recorded that decolorization efficiency of MG by *P. aeruginosa* and *B. cereus* was the best when incubated at 37 °C for 72 h and recorded 91.5% and 98.5%, respectively. It was reported by [34] that 37 °C was ideal for decolorization of Reactive brilliant Blue by *Candida*, also they found that increase temperature more than 37 °C inhibited the decolorization of Reactive brilliant Blue by tested yeast strains. Which might due to the harmful effects of high temperature on enzymes responsible for decolorization [35, 36] reported that maximum decolourization percentage of Remazol Black-B dye by *Kluyveromyces marxianus* IMB3 was 98%, which achieved at 37 °C.

The selected isolate *R. mucilaginosa* in this work has no activity at the concentration 1000 mg/L MG dye as it was very toxic and inhibited the growth of yeast and enzymes even after 168 h. Complete decolorization 100% was done at a concentration of 50 mg/L and it was 99.7% at MG dye concentration 100 mg/L [31]. discovered that as dye concentration increased, the percentage of dye decolorization for malachite green declined by *B. cereus* and the maximum decolourization percentage was 91.2% at dye concentration 100 mg/L [34]. recorded that the decolorization efficiency of Reactive brilliant Blue by *R. glutinis*,* Candida sphaerica* and *C. utilis* was decreased at the dye concentration more than 200 mg/L. This might explain by harmful effects of high concentration of chemical dyes on the yeast cell growth which causes the reduction in the amount of yeast biomass used in dye biodegradation. Also, high concentration of dye could deactivate enzymes responsible for degradation of dye [26, 37] reported that *Flavobacterium* sp. had excellent MG decolorization performance at 150–250 mg/L MG with nearly complete decolorization over a 12 h incubation time. When MG concentrations were raised to 300 mg/L and 350 mg/L after a 12 h incubation time, respectively the decolorization percentage decreased to approximately 47 ± 1% when the MG concentration was 500 mg/L [38, 39]. and others have shown that the dye biodegradation process depends on the concentration of the dye and the ideal concentration of dye would be different between different microbial species and in general the maximum decolorization efficiency has been observed at moderate dye concentrations.

At the end of optimization process the cells of test yeast strain was examined under light microscope and they were colorless at 100% decolorization percentage under optimized conditions. This indicated that the mechanism of decolorization of MG dye was biodegradation [40]. mentioned that bioremediation of dyes by microorganisms could be carried out by different mechanisms included biosorption, detoxification, bio-degradation, bioaccumulation and enzymatic mineralization such as Manganese peroxidase, lignin peroxidase and laccase enzymes.

### The GC-MS analysis

Recorded thirteen metabolites produced from the biodegradation of MG dye by *R. mucilaginosa* AUMC13567. The produced degradation metabolites including different compounds containing easily biodegradable functional groups of the ring substituents such as phenolic, alcohol, amino, or other that produced from aromatic cleavage, which was in agreement with [41–43].

FTIR spectrum of MG molecules after 12 h of cultivation with yeast strain shows appear of new functional groups in comparing to the spectrum of undegraded MG molecules at wave length 2956.18, 2924.37 and 2853.54 which refer to C–H stretch (alkyl groups) and disappear of peaks at wave length 1614.81 cm^− 1^ refers to C = C stretch (aromatic ring), or C = N stretch and 1585.7 cm^− 1^ which refers to aromatic C = C stretch or N–H bend. In addition to reduced number of peaks at wave length less than 1000 cm^− 1^ that refers to aromatic rings which means change the aromatic structure of MG molecules due to the biodegradation process that performed by the yeast strain [44] and [45]. suggested that the first stage of MG dye molecule decomposition occurs from cleavage of the bond between the central carbon, by attack of OH, leading to the production of aromatic compounds [45]. discovered that phenols, benzophenones, and derivatives of benzoic acid were among the aromatics produced by the breakdown of malachite green dye. These aromatics were then transformed into hydroquinone and p-benzoquinone. These compounds were broken down into aliphatic carboxylic acids [46]. recorded that *Pseudomonas veronii* degrade MG dye under the static condition to Methanone [4-(dimethylamino) phenyl] phenyl (m/z 225); Phosphinic acid, bis [p- (dimethylamino)phenyl], methyl ester (m/z 318), (E)-2-Hydroxy-4’-dimethylamino-stilbene (m/z 239) and Benzylaniline (m/z 183). Revealed the following observations where the peaks in the display the existence of –OH, - NH, -C-H (amides and amines).

Due to the results of cytotoxicity test of MG dye before and after biodegradation by the selected yeast strain on three human cell lines (CaCo-2, Hep-2, HSF) they showed a minimum toxicity of MG metabolites after biodegradation. In previous study in our laboratory we test the same strain of yeast [47] this stain showed good results during the study of the detoxification of 0.25 mg/l Malachite Green dye by *Rhodotorula mucilaginosa* AUMC13567 on twenty-four *Clarias gariepinus* fish recorded the total bacterial count on the skin, gills, and intestine in Group (exposed to Malachite Green degradation metabolites) had significantly bacterial load lower than those of Group (exposed to Malachite Green dye) and the Control Group. Also, The ***Rhodotorula mucilaginosa*** AUMC13567was showed highly efficiency to reduce the phytotoxic effect of MG and the metabolites of the biodegraded dye were nontoxic to seeds of wheat (*T. aestivum*), sorghum (*Sorghum bicolor*), maize (*Zea mays*), and radish (*Raphanus sativus*) [21].

## Conclusion

This study demonstrates that the best conditions for 100% decolorization of MG dye at concentration 50 mg/L by *R. mucilaginosa* AUMC13567 were cultivation on modified YME medium composed of distilled water with 3% glucose under shaking conditions at 150 rpm, 37˚C for 12 h. And biodegradation metabolites found to be saved on three human cell lines, including colorectal cancer, head and neck cancer, and healthy skin fibroblast. Furthermore, scale-up studies under optimized conditions should be investigated in the future to assess the viability of implementing this biodegradation process in industrial wastewater treatment. Examining the long-term ecological effects and biodegradation efficacy of mixed dye pollutants or actual textile effluents might yield valuable information for environmental applications.

## Electronic supplementary material

Below is the link to the electronic supplementary material.

Supplementary Material 1

## Data Availability

No datasets were generated or analysed during the current study.

## References

[1] Upadhyay N. Involvement of microorganisms in the biodegradation of synthetic dyes of textile waste. Acta Sci Microbiol. 2023;6(2):80–5.

[2] Al-Tohamy R, Ali SS, Li F, Okasha KM, Mahmoud YAG, Elsamahy T, Sun J. A critical review on the treatment of dye-containing wastewater: ecotoxicological and health concerns of textile dyes and possible remediation approaches for environmental safety. Ecotoxicolog Envir Safet. 2022;231:113160. 10.1016/j.ecoenv.2021.113160 35026583

[3] Chandanshive V, Kadam S, Rane N, Jeon BH, Jadhav J, Govindwar S. In situ textile wastewater treatment in high rate transpiration system furrows planted with aquatic macrophytes and floating phytobeds. Chemosph. 2020;252:126513. 10.1016/j.chemosphere.2020.126513 32203784

[4] Abu-Hussien SH, Hemdan BA, Alzahrani OM, Alswat AS, Alatawi FA, Alenezi MA, El-Sayed SM. Microbial degradation, spectral analysis and toxicological assessment of malachite green dye by *streptomyces exfoliatus*. Molecl. 2022;27(19):6456. 10.3390/molecules27196456 PMC9572514 36234993

[5] Garcia-Segura S, Bellotindos LM, Huang YH, Brillas E, Lu MC. Fluidized-bed Fenton process as alternative wastewater treatment technology. A review. J Taiw Institu Chem Engin. 2016;67:211–25.

[6] Sahoo S, Kumar A, Upadhyay A. How do green knowledge management and green technology innovation impact corporate environmental performance? Understanding the role of green knowledge acquisition. Busin Strate Envir. 2023;32(1):551–69.

[7] Ruscasso F, Cavello I, Curutchet G, Cavalitto S. Antarctic yeasts: potential use in a biologic treatment of textile Azo dyes. Bioresourc Bioprocess. 2022;9(1):18. 10.1186/s40643-022-00507-5 PMC10991636 38647816

[8] Samsami S, Mohamadizaniani M, Sarrafzadeh MH, Rene ER, Firoozbahr M. (2020). Recent advances in the treatment of dye-containing wastewater from textile industries: Overview and perspectives. Proc. Safe. and Environm. Protect., 143, 138–163.

[9] Al-Tohamy R, Kenawy ER, Sun J, Ali SS.)_a_. Performance of a newly isolated salt-tolerant yeast strain *Sterigmatomyces halophilus* SSA-1575 for Azo dye decolorization and detoxification. Frontier Microbio. 2020;11:1163. 10.3389/fmicb.2020.01163 PMC7300265 32595618

[10] Al-Tohamy R, Sun J, Fareed MF, Kenawy ER, Ali SS.)_b_. Ecofriendly biodegradation of reactive black 5 by newly isolated *Sterigmatomyces halophilus* SSA1575, valued for textile Azo dye wastewater processing and detoxification. Scient Repor. 2020;10(1):12370. 10.1038/s41598-020-69304-4 PMC7378048 32704008

[11] Vaidya K, Konde PU. Decolorization of malachite green by *Sporotrichum pulverulentum*. J Ind Pollut Control. 2008;24:133–7.

[12] Li Z, Li C, Cheng P, Yu G. *Rhodotorula mucilaginosa*—alternative sources of natural carotenoids, lipids, and enzymes for industrial use. Heliyon; 2022. e11505. 10.1016/j.heliyon.2022.e11505 PMC9676536 36419653

[13] Gualberto NC, Nogueira JP, da Silva ADS, Barbosa PF, Matos CMS, Rajan M, Narain N. Optimization of the biotechnological process using *Rhodotorula mucilaginosa* and acerola (*Malpighia emarginata L.*) seeds for the production of bioactive compounds. LWT. 2022;160:113190.

[14] Ge Y, Huang K, Xie W, Xu C, Yao Q, Liu Y. Effects of Rhodotorula mucilaginosa on the immune function and gut microbiota of mice. Fron Fung Biolo. 2021;2:705696. 10.3389/ffunb.2021.705696. 10.3389/ffunb.2021.705696 PMC10512290 37744147

[15] Maoka T. Carotenoids as natural functional pigments. J Natur Medicin. 2020;74(1):1–16. 10.1007/s11418-019-01364-x PMC6949322 31588965

[16] Eman Mostafa M. Fungal and yeast carotenoids. Rev J Yeas Fung Resear. 2019;10(2):30–44.:10.5897/JYFR 2019.0192.

[17] Mostafa AA, Abou-Zeid AM, El-Zaher EHFA, Arif DM. Bitreatment of industrial oil waste water by free and immobilized Rhodotorula mucilaginosa 2 and Candida utilis. Advanc Biologic Resear. 2015;9(4):271–80.

[18] Awadeen NA, Eltarahony M, Zaki S, Yousef A, ElAssar S, ElShall H. Fungal carbonatogenesis process mediates zinc and chromium removal via statistically optimized carbonic anhydrase enzyme. Microb Cell Fact. 2024;23:236. 39192338 10.1186/s12934-024-02499-7 PMC11350955

[19] Ren S, Guo J, Zeng G, Sun G. Decolorization of triphenylmethane, Azo, and anthraquinone dyes by a newly isolated *Aeromonas hydrophila* strain. Appl Microbiolo Biotechnolo. 2006;72:1316–21. 10.1007/s00253-006-0418-2 16622679

[20] Wickerham LJ. *Taxonomy of yeasts* (No. 1029). US department of agriculture; 1951.

[21] Allam NN, Mohamed EM, Ali M, Nassar SM. (2023). Screening of yeast ability to decolorization and completely biodegradation of malachite green textile dye and investigate their phytotoxicity. Bulletin of Pharmaceutical Sciences Assiut University. 11, Volume 47, Issue 1, June 2024, Page 179–195.

[22] Dhanve RS, Shedbalkar UU, Jadhav JP. (2008). Biodegradation of diazo reactive dye Navy Blue HE2R (Reactive Blue 172) by an isolated Exiguobacterium sp. RD3. Biotechno. and Bioproc. Engin., 13, 53–60.

[23] Daneshvar N, Ayazloo M, Khataee AR, Pourhassan M.)_a_. Biological decolorization of dye solution containing malachite green by microalgae *Cosmarium* Sp. Biores Techno. 2007;98(6):1176–82. 10.1016/j.biortech.2006.05.025 16844368

[24] Daneshvar N, Khataee AR, Rasoulifard MH, Pourhassan M.)_b_. Biodegradation of dye solution containing malachite green: optimization of effective parameters using Taguchi method. J Hazar Mater. 2007;143(1–2):214–9. 10.1016/j.jhazmat.2006.09.016 17052836

[25] Parshetti G, Kalme S, Saratale G, Govindwar S. (2006). Biodegradation of malachite green by *Kocuria rosea* MTCC 1532. Acta Chim Slov, 53(4).

[26] Jadhav JP, Parshetti GK, Kalme SD, Govindwar SP. (2007). Decolourization of azo dye methyl red by *Saccharomyces cerevisiae* MTCC 463. Chemos., 68(2), 394–400. 10.1016/j.chemosphere.2006.12.087 17292452

[27] Demet Ç, Göonöul D. Decolourization of reactive dyes by mixed cultures isolated from textile effluent under anaerobic conditions. Enzyme Microbiol Technol. 2006;38:926–30.

[28] Oranusi NA, Ogugbue CJ. Effect of pH and nutrient starvation on biodegradation of Azo dyes by *Pseudomonas* Sp. J Appl Scienc Envir Managem. 2005;9(1):39–43.

[29] Skehan, P., Storeng, R., Scudiero, D., Monks, A., McMahon, J., Vistica, D.,… Boyd,M. R. (1990). New colorimetric cytotoxicity assay for anticancer-drug screening. JNCI, 82(13), 1107–1112. 10.1093/jnci/82.13.1107 2359136

[30] Yang, Q., Zhang, H., Li, X., Wang, Z., Xu, Y., Ren, S.,… Wang, H. (2013). Extracellular enzyme production and phylogenetic distribution of yeasts in wastewater treatment systems. Bioresour. Technolo., 129, 264–273. 10.1016/j.biortech.2012.11.101 23261999

[31] Alshehrei F. Optimization of malachite green and crystal Violet decolorization by *Bacillus cereus* and *Pseudomonas earoginosa*. Ann Biol Res. 2018;9:1–8.

[32] Zuraida MS, Nurhaslina CR, Halim KK. (2013). Adsorption of colour from Batik effluent by bacterial, *Lactobacillus Delbruckii* and its growth. Busin. Engin. and Industria. Appli. Colloq. (BEIAC) (pp. 574–579).

[33] Bamforth SM, Singleton I. Bioremediation of polycyclic aromatic hydrocarbons: current knowledge and future directions. J Chemi Techn Biotechno: Inter Resear Proc Envir Clean Techn. 2005;80(7):723–36.

[34] Ashour S, Abo-Ghalia H. Optimization of environmental parameters on the decolorization of reactive brilliant blue dye by yeast isolates from textile effluent. J Scient Resear Scie. 2015;32(1):122–46.

[35] Shah MP, Patel KA, Nair SS, Darji AM. (2013). Optimization of environmental parameters on microbial degradation of reactive black dye. J Bioremedia Biodegrad, 4(3).

[36] Meehan C, Banat IM, McMullan G, Nigam P, Smyth F, Marchant R. Decolorization of remazol Black-B using a thermotolerant yeast, Kluyveromyces Marxianus IMB3. Envir Internation. 2000;26(1–2):75–9. 10.1016/s0160-4120(00)00084-2 11345742

[37] Maniyam MN, Gunalan P, Azman HH, Abdullah H, Yaacob NS. Enhancing biodecolorization of malachite green by Flavobacterium Sp. through monothetic analysis. J Indian Chem Soc. 2024;101:101452.

[38] Khalid A, Arshad M, Crowley DE. Accelerated decolorization of structurally different Azo dyes by newly isolated bacterial strains. Appl Microbiolo Biotechnolo. 2008;78:361–9. 10.1007/s00253-007-1302-4 18084755

[39] Sponza DT, Işık M. (2005). Reactor performances and fate of aromatic amines through decolorization of Direct Black 38 dye under anaerobic/aerobic sequentials. Proc. Biochemist., 40(1), 35–44.

[40] Hameed BB, Ismail ZZ. (2018). Decolorization, biodegradation and detoxification of reactive red Azo dye using non-adapted immobilized mixed cells. Biochem Eng J; 137.

[41] Zubbair NAA, Ajao AT, Adeyemo EO, Adeniyi OD. Biodegradation of malachite green by white-rot fungus, pleurotus pulminarious. Egypt Acad J Biol Sci G Microbiolo. 2020;12(1):79–90.

[42] Wanyonyi WC, Onyari JM, Shiundu PM, Mulaa FJ. Biodegradation and detoxification of malachite green dye using novel enzymes from *bacillus cereus* strain KM201428: kinetic and metabolite analysis. Energy Procedia. 2017;119:38–51.

[43] Paszczynski A, Goszczynski S, Crawford DL, Crawford RL. Influence of aromatic substitution patterns on Azo dye degradability by Streptomyces spp. And *Phanerochaete Chrysosporium*. Appl Enviromental Microbiol. 1992;58:11. 10.1128/aem.58.11.3605-3613.1992 PMC183151 1482183

[44] Xie Y, Wu K, Chen F, He J, Zhao J. Investigation of the intermediates formed during the degradation of malachite green in the presence of Fe ^3+^ and H_2_O_2_ under visible irradiation. Resear Chemic Intermediat. 2001;27:237–48.

[45] Oturan MA, Guivarch E, Oturan N, Sirés I. Oxidation pathways of malachite green by Fe^3+^-catalyzed electro-Fenton process. Appl Cataly B: Envir. 2008;82(3–4):244–54.

[46] Song J, Han G, Wang Y, Jiang X, Zhao D, Li M, Mu Y. Pathway and kinetics of malachite green biodegradation by *Pseudomonas veronii*. Sci Rep. 2020;10:4502. 10.1038/s41598-020-61442-z. 32161360 10.1038/s41598-020-61442-z PMC7066194

[47] Nassar S, Sayed AH, Nafady NA, Ali MM, Mohamed EM. (2024). Bioremediation of the toxic effects induced by the malachite green dye in *Clarias gariepinus* using *Rhodotorula mucilaginosa* MH298827. Scientific African. 26, e02496.

