# Supplementary material for: Bioremediation of malachite green dye toxicity under optimized conditions by Rhodotorula mucilaginosa AUMC13567
Source: BMC Biotechnol. 2025 May 17;25:39. doi: 10.1186/s12896-025-00977-3 (PMC12084967; doi:10.1186/s12896-025-00977-3)
Supplement: Supplementary file 1 — Supplementary Material 1 [file 12896_2025_977_MOESM1_ESM.docx]

**Table (S1): Cytotoxicity of Malachite Green before and after degradation by *Rhodotorula mucilaginosa* _AUMC13567_ on Colorectal cancer (CaCo-2) cell line.**

| **Cell line** | **Malachite Green** | | **Degradable Malachite Green** | |
| --- | --- | --- | --- | --- |
| **Blank** | 0.082 | | 0.079 | |
| **Treatments** | **Data** | **Viability** | **Data** | **Viability** |
| **Control** | 4.6±0.1 ^c^ | 100±0.0 ^c^ | 4.6±0.1 | 100±0.0 |
| **0.01** | 4.7±0.0 ^c^ | 100±0.5 ^c^ | 4.6±0.1 | 99.0±1.6 |
| **0.25** | 1.9±0.1 ^b^ | 40.7±1.5 ^b^ | 4.6±0.0 | 98.5±0.2 |
| **5** | 0.1±0.0 ^a^ | 1.1±0.3 ^a^ | 4.5±0.0 | 97.7±1.1 |
| **50** | 0.0±0.0 ^a^ | 0.7±0.3 ^a^ | 4.5±0.1 | 96.4±1.8 |
| **100** | 0.0±0.0 ^a^ | 0.4±0.0 ^a^ | 4.4±0.1 | 96.2±1.1 |
| **F-value** | 3872*** | 5511*** | - | - |

**Table (S2): Cytotoxicity of Malachite Green before and after decolorization by *Rhodotorula mucilaginosa* _AUMC13567_ on Head and neck cancer cell line.**

| **Cell line** | **Malachite Green** | | **Degradable Malachite Green** | |
| --- | --- | --- | --- | --- |
| **Blank** | 0.069 | | 0.074 | |
| **Treatments** | **Data** | **Viability** | **Data** | **Viability** |
| **Control** | 5.3±0.0^c^ | 100±0.0^c^ | 5.4±0.0^b,c^ | 100.9±0.5^b^ |
| **0.01** | 5.3±0.1^c^ | 100±2.6^c^ | 5.4±0.1^c^ | 101.4±0.9^b^ |
| **0.25** | 2.4±0.0^b^ | 45.6±0.7^b^ | 5.4±0.0^c^ | 100±0.0^b^ |
| **5** | 0.2±0.0^a^ | 4.4±0.5^a^ | 5.4±0.1^b,c^ | 100.1±0.9^b^ |
| **50** | 0.2±0.1^a^ | 3.1±0.9^a^ | 5.3±0.0^a,b^ | 97.8±0.1^a^ |
| **100** | 0.2±0.0^a^ | 2.9±0.7^a^ | 5.2±0.1^a^ | 96.3±1.0^a^ |
| **F-value** | 1444 ^***^ | 1490 ^***^ | 7^***^ | 8^***^ |

**Table (S3): Cytotoxicity of Malachite Green before and after degradation by *Rhodotorula mucilaginosa* _AUMC13567_ on healthy Human Skin Fibroblast (HSF) cell line.**

| **Cell line** | **Malachite Green** | | **Degradable Malachite Green** | |
| --- | --- | --- | --- | --- |
| **Blank** | 0.082 | | 0.090 | |
| **Treatments** | **Data** | **Viability** | **Data** | **Viability** |
| **Control** | 5.4±0.1^d^ | 100±0.0^e^ | 5.4±0.0^b,c^ | 100±0.0^b,c^ |
| **0.01** | 5.4±0.0^d^ | 99.7±0.2^e^ | 5.4±0.0^c^ | 101.7±0.5^c^ |
| **0.25** | 1.6±0.0^c^ | 28.8±0.5^d^ | 5.3±0.0^b^ | 99.6±0.3^b^ |
| **5** | 0.3±0.0^b^ | 5.6±0.7^c^ | 5.3±0.0^a,b^ | 99±0.6^a,b^ |
| **50** | 0.1±0.0^a^ | 2.5±0.3^b^ | 5.3±0.0^a,b^ | 98.5±0.8^a,b^ |
| **100** | 0.0±0.0^a^ | 0.5±0.4^a^ | 5.2±0.1^a^ | 97.4±1.0^a^ |
| F-value | 5062*** | 13408 *** | 6** | 6** |
